# The Role of Glutathione in Prevention of COVID-19 Immunothrombosis: A Review

**DOI:** 10.31083/j.fbl2803059

**Published:** 2023-03-20

**Authors:** Ira Glassman, Nghia Le, Mercedeh Mirhosseini, Cheldon Alcantara, Aamna Asif, Anabel Goulding, Shafi Muneer, Manpreet Singh, Jeremiah Robison, Frederick Guilford, Vishwanath Venketaraman

**Affiliations:** 1 College of Osteopathic Medicine of the Pacific, Western University of Health Sciences, Pomona, CA 91766, USA; 2 Department of Emergency Medicine, Corona Regional Medical Center, Corona, CA 92882, USA; 3 Department of Emergency Medicine, St. Barnabas Hospital Health System, Bronx, NY 10457, USA; 4 Your Energy System, Palo Alto, CA 94301, USA

**Keywords:** glutathione, GSH, COVID-19, SARS-CoV-2, immunothrombosis, HIV, diabetes, microclot, thrombosis

## Abstract

Immunothrombosis has emerged as a dominant pathological process exacerbating morbidity and mortality in acute- and long-COVID-19 infections. The hypercoagulable state is due in part to immune system dysregulation, inflammation and endothelial cell damage, as well as a reduction in defense systems. One defense mechanism in particular is glutathione (GSH), a ubiquitously found antioxidant. Evidence suggests that reduction in GSH increases viral replication, pro-inflammatory cytokine release, and thrombosis, as well as decreases macrophage-mediated fibrin removal. The collection of adverse effects as a result of GSH depletion in states like COVID-19 suggest that GSH depletion is a dominant mechanism of immunothrombosis cascade. We aim to review the current literature on the influence of GSH on COVID-19 immunothrombosis pathogenesis, as well as the beneficial effects of GSH as a novel therapeutic for acute- and long-COVID-19.

## 1. Introduction

The COVID-19 pandemic has claimed roughly 6.7 million lives as of January 2023 per the World Health Organization. SARS-CoV-2 is an enveloped positive-sense single-stranded RNA virus that spreads through direct, indirect, or close contact with infected hosts by respiratory droplets. The initial entry of the virus into human cells was found to be via the SARS-CoV-2 spike (S) protein binding to the cell surface receptor angiotensin-converting-enzyme-2 (ACE2) expressed by type II pneumocytes [1]. Binding and entry leads to a cascade of steps resulting in inflammation and ultimately systemic disease [2].

While the disease severity can range from mild to severe, commonly reported symptoms include loss of smell, fever, fatigue, and dizziness. Severely affected patients exhibit acute respiratory distress syndrome (ARDS) leading to oxygen deficiency [3]. Researchers have identified one key pathologic component associated with infection severity and course, termed immunothrombrosis. Immunothrombosis describes the interconnectivity between the body’s natural coagulation processes and the innate immune system. The overall effect is the formation of thrombi within small and large blood vessels. In states of hyperinflammation, like COVID-19, patients are marked by a dysregulated and hyperactive innate immune system as well as a hypercoagulable state. The immunothrombosis cascade normally depends on a number of cytokines and inflammatory mediators which is intended to provide a protective role. When dysregulated, the process can lead to the development of excessive cytokine release, termed cytokine storm [4]. After observing the cardiopulmonary tissue of a subset of patients with severe COVID-19 who also developed ARDS, immunothrombosis was identified as a significant contributing factor to these patients critical conditions [5,6]. Specifically, the presence of amyloid fibrin microclots which are resistant to fibrinolysis and found microvessels in both acute and Long-COVID-19 [6]. Immunothrombosis is dependent on platelet aggregation and mediators of platelet aggregation, one of which is the ubiquitous antioxidant glutathione in its reduced form (GSH). Studies have shown that GSH may act as an inhibitor of platelet aggregation and in many cases of COVID-19, a deficiency in GSH has been observed [7,8].

GSH is an important antioxidant that naturally works to limit the levels of dangerous reactive oxygen species (ROS). Decreased levels of GSH and elevated levels of ROS have been associated with COVID-19 pathogenesis [9]. COVID-19 is also associated with elevated levels of D-dimer, a breakdown product of fibrin clots, and has been used as a specific biomarker to indicate severity of COVID-19 [10]. With exacerbated oxidative stress and cytokine storm, increased levels of interleukin-6 (IL-6) and decreased levels of interferon-*β* (IFN-*β*) are thought to contribute to worsening infection and are also associated with decreased levels of GSH [11]. There also exists a negative correlation between macrophage function and GSH levels, which may serve a key role in the dysregulation of immunothrombosis [12].

The significance of these findings identify an additional pathologic component of COVID-19 and also serve to identify novel adjunctive therapies. Confirming what role GSH plays in the pathophysiology of COVID-19 and immunothrombosis can serve as a scaffold for future therapeutic research. We aim to review the role of GSH in COVID-19 pathogenesis, immunothrombosis, and immune system dysregulation.

## 2. Search Strategy and Selection Criteria

This article is a comprehensive review investigating the role of glutathione in the COVID-19 immunothrombosis cascade. Information was obtained using PubMed and NCBI databases from December 2022 to February 2023. Search results included terms such as: “Glutathione”, “COVID-19”, “SARS-CoV-2”, “Immunothrombosis”. Search terms specific to respective subsections included “glutathione peroxidase”, “oxidized glutathione”, “interleukin 6”, “transforming growth factor beta”, “macrophage”, “D-dimer”, “cytokines”, “reactive oxygen species” and “thrombosus”. Research included in this article were selected based on quality and significance of results. Review articles included were chosen based on comprehensiveness of the topic of interest. Exclusion criteria included non-relevance, poor sample size, and inconclusive data and significance. A total of 226 articles were identified, 122 were excluded based on exclusion criteria, and 104 articles were included in qualitative synthesis.

## 3. Immunothrombosis Overview

Thrombosis is the formation of a clot inside blood vessels which leads to partial or complete vessel occlusion, preventing bleeding after vessel injury. Clots are formed when vascular damage activates tissue factor (TF), which leads to recruitment of platelets and initiation of the coagulation cascade. Finally, the clot is stabilized using fibrin and thrombin. TF is continuously delivered to the clot to participate in its growth [13]. Interestingly, immune cells such as neutrophils and monocytes can also interact with platelets and the coagulation cascade to induce clot formation in blood vessels [1]. Neutrophils release neutrophil extracellular traps (NETs) which bind to and activate multiple coagulation factors, attract platelets, and bind TF to amplify the clotting cascade [1]. Monocytes release microparticles that express intravascular TF to further activate coagulation and create microthrombi matrices which immune cells use to assist them in recognition, containment, and destruction of pathogens in a process termed immunothrombosis [14]. Research shows that intravascular TF may also be found in neutrophils, eosinophils, and platelets [14].

**Fig. 1.**
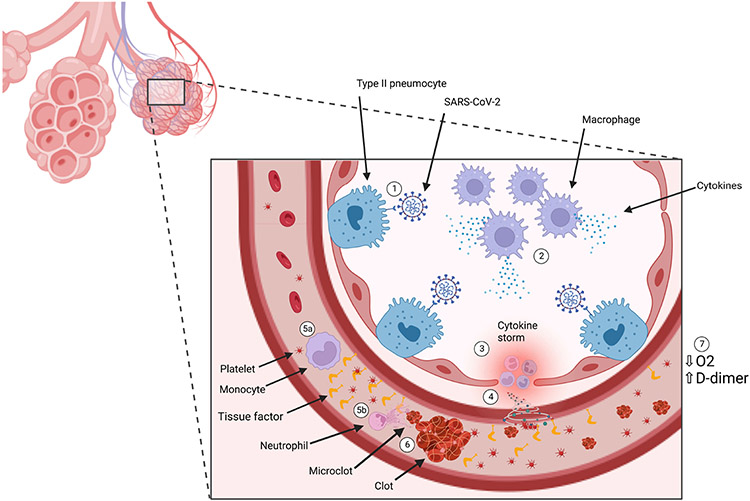
Immunothrombosis induction. (1) SARS-CoV-2 binds to ACE-2 receptor on type-2 pneumocyte cells. (2) Infection with SARS-CoV-2 results in recruitment of immune cells, including macrophages, which release cytokines. (3) Cytokines recruit more immune cells which produce excess cytokines, otherwise known as a cytokine storm. (4) The cytokine storm results in endothelial inflammation and damage, releasing tissue factor (TF) into circulation. (5) Cytokines recruit (a) monocytes, which release microparticles expressing intravascular TF, and (b) neutrophils, which release neutrophil extracytoplasmic traps (NETs). (6) The combination of TF and NETs activate immunothrombosis, generating clots and microclots. (7) Clotting and impaired blood flow results in impaired oxygen delivery and elevated D-dimer levels.

Hyperinflammatory states such as those seen in COVID-19 can trigger immunothrombosis [15]. Severe COVID-19 induces extensive pulmonary inflammation, triggering a macrophage activation syndrome (MAS)-like event with proinflammatory cytokines, macrophage and lymphocyte recruitment, and subsequent endothelial damage [15]. Endothelial dysfunction is associated with decreased bioavailability of nitric oxide (NO) due to an increase in ROS, with a similar physiological change seen in cases of decreased GSH pools [16]. The decrease in NO increases the expression of transcription factor nuclear factor kappa B (NF-*κ*B) which serves to increase synthesis of cytokines and adhesion molecules. The adhesion molecules promote binding and transmigration of leukocytes and increase cell-cell interactions important for platelet function [17]. In advanced atherosclerotic lesions, decreased NO is associated with thrombus formation by increasing platelet adhesion and aggregation [18]. The endothelial damage, release of TF and intravascular TF, and elevated proinflammatory cytokines trigger immunothrombosis in the pulmonary microvasculature, yielding microclots [15]. The coagulation cascade can be progressively exacerbated by development of local hypoxia, creating a positive thromboinflammatory feedback loop within the small vessels of the lungs, seen in Fig. 1 [15]. Post-acute sequelae of COVID (PASC), otherwise known as Long-COVID, can be attributed to these microclots which block transport of erythrocytes in capillaries and therefore oxygen delivery [6].

Severe COVID-19 is marked by high levels of D-dimer, a fibrin byproduct that can indicate prevalence of thrombotic formation. Severe COVID-19 patients experience a 9-fold increase in the prevalence of alveolar-capillary microthrombi when compared to patients with influenza [19]. It is important to identify therapies to prevent the thromboembolic complications of COVID-19. Early reports on the use of low molecular weight heparin (LMWH) for severe COVID-19 cases with elevated D-dimer have demonstrated a reduction in mortality [20]. However, according to the American College of Chest Physicians (CHEST) guidelines, thromboprophylaxis therapy is only recommended for hospitalized patients. Unfortunately, patients remain at an increased risk for thromboembolic complications even after resolution of acute infection. Patients with new or persistent symptoms following the acute phase of COVID-19 should be followed to rule out thromboinflammatory disease [21]. A systematic literature review conducted by Overton *et al.* [21] revealed that there is little global standardization of thromboembolism reporting and a low rate of acute COVID-19 vasculopathy detection, suggesting that current diagnostic methods for identifying pulmonary vascular disease may not be sufficient. Further development of tools to detect abnormalities in pulmonary vasculature and differentiate between thromboinflammatory disease from thromboembolic disease is needed [21]. Development of therapeutics to prevent the hyperthromboinflammatory state seen in COVID-19 is also of high value.

## 4. GSH Overview

Glutathione (GSH) is a low molecular weight antioxidant present in nearly all cells that functions to limit the impact of oxidative stress on vital cellular components such as lipids, proteins, and DNA through redox reactions [22]. GSH can be found in the mitochondria, nucleus, and cytosol of cells, as well as plasma and extracellular spaces like the fluid which lines pulmonary alveoli [22]. GSH is increased when the body is exposed to oxidants and electrophiles and, when decreased, yields a vulnerable state for some diseases [22]. There is an association between chronic pulmonary inflammation and low GSH levels, exemplified in cystic fibrosis patients who produce less GSH and smokers who have been found to have lower levels of GSH [22].

Glutathione has been found to be directly related to thrombosis and thrombotic events through many mechanisms, generally related to platelet attenuation and activity on the coagulation cascade [23,24]. GSH is utilized by glutathione peroxidase (GPX) to reduce free hydrogen peroxide and lipid hyperoxides. Jin *et al.* [25] found that knock-out of plasma GPX, GPX-3, resulted in increased platelet-dependent thrombosis in *murine* models. A deficiency in GPX-3 reduces metabolism of ROS, partly due to a reduction in NO which results in increased platelet adhesion and aggregation, as mentioned earlier [25]. The antioxidant properties of GPX-3 are also thought to protect against post-translational modifications of fibrinogen by ROS and NO-derived oxidants resulting in increased thrombogenicity [26]. Furthermore, Dayal *et al.* [27] found that overexpression of the most abundant GPX, GPX-1, served a protective role from platelet hyperactivity and age-dependent increased susceptibility to venous thrombosis after inferior vena cava ligation in *murine* models.

Thomas *et al.* [28] evaluated the relationship between GSH in unstimulated platelets from diabetic versus control subjects, finding that diabetic subjects have lower GSH levels in their platelets than control subjects. They also measured the amount of thromboxane A2 (TXA2) produced by activated platelets in diabetic versus control subjects, finding that diabetic subjects produce more TXA2 than control subjects. TXA2 stimulates the activation of new platelets, increases platelet aggregation, and is a vasoconstrictor. In states of increased oxidative stress, there is increased oxidized GSH (GSSG) [29]. Essex *et al.* [30] have found that decreased GSH or a mixture of GSSG and GSH can increase platelet aggregation. Furthermore, they found that GSSG alone is able to increase platelet aggregation [30]. They suggested an agonist-induced mechanism whereby the addition of GSSG to platelets generated sulfhydryls in the *β* subunit of the *α*_IIb_*β*_3_ fibrinogen receptor [30]. These findings further suggest a connection between low GSH levels and prothrombotic platelet activity [28].

Recently, Wang *et al.* [31] discovered another connection between GSH and platelet aggregation. Protein disulfide isomerase (PDI) is an endoplasmic reticulum (ER)-resident oxioreductase found in platelets that is critical for platelet aggregation. PDI is oxidized by ER oxidoreductin-1*α* (Ero1*α*) which has been found to contribute to redox-controlled remodeling of *α*IIb*β*3. Wang *et al.* [31] found that oxidized PDI regulates platelet aggregation in a GSH-dependent manner. They found that in plasma with GSH depletion, neither oxidized nor reduced PDI promoted platelet aggregation. Furthermore, they found that reduced PDI and Ero1*α* oxidized GSH to GSSG and that the optimal GSH:GSSG ratio for platelet aggregation is 5:1 [31]. PDI inhibitors are already being investigated as novel antithrombotic therapeutics and these findings highlight the importance of GSH in platelet aggregation, as well as a potential therapeutic.

Pacchiarini *et al.* [7] evaluated the effect of GSH on platelet functions. They found GSH concentrations of 3 mM or 10 mM were able to modify platelet aggregation, TxB2 production, and PDGF release by platelets. Of these three parameters, TxB2 production was significantly reduced at both doses, PDGF release was significantly reduced at 10 mM, and collagen-induced platelet aggregation was not significantly reduced [7]. Thomas *et al.* [32] found that GSH is able to inhibit human platelet aggregation induced by adenosine diphosphate (ADP), collagen, and arachidonic acid. Together, these findings suggest that GSH may serve to inhibit platelet activation when administered exogenously. Furthermore, activated platelets produce ROS through NADPH oxidase (NOX) signaling and induction of mitochondrial dysfunction. This sets up a cycle of repeated ROS production, platelet activation, adhesion, and recruitment which contributes to the prothrombotic risk seen in inflammatory conditions like COVID-19 [33].

Studies on patients that suffered both atherosclerotic and cardioembolic stroke found that low plasma levels of GSH could be an independent risk factor for stroke severity. During acute ischemia following a stroke, tissue recovery is based on antithrombotic activity of the body, as well as resistance to oxidative stress caused by the ischemia. GSH is a low molecular weight aminothiol, protecting other cellular thiols from oxidative damage, and helping reduce reactive oxygen species the cell is exposed to. In addition to this it is heavily involved in platelet attenuation [23].

In experimental stroke models, platelet aggregometry and thromboelastography (ROTEM) demonstrated activity of vWF that cross links platelets in arterial thrombi, the end product of the coagulation cascade that promotes clot formation [34]. Diabetic patients are known to have a multifactorial hypercoagulable state related to increased inflammatory markers, and increased platelet aggregation (and indeed, an increased risk for downstream clinical effects such as stroke, heart attack, and other thrombotic events). GSH levels have not only been found to be low in these patients, but replacement with N-acetylcysteine (NAC), a synthetic precursor for GSH, have been found to reduce thrombin activation and platelet activation [24]. Wang *et al.* [35] found that the percentage of circulating blood-platelet leukocyte aggregates (PLAs) were significantly elevated in diabetes, with platelets having lower GSH and GPX-1 levels. These findings were also associated with increased methylglyoxal (MG), which is normally cleared by GSH. They found that administration of NAC enhanced platelet GSH and GSH-dependent MG elimination, as well as corrected levels of GPX-1 [35].

The depletion of GSH may also allow viruses to replicate, and therefore is a major factor in the pathology of COVID-19 [36]. When SARS-CoV-2 invades the body it elevates proinflammatory markers such as IL-6 and TGF-*β*, which prevent the formation of GSH [36]. COVID-19 also depletes GSH through intracellular radical generation and inhibition of BRCA1, a DNA damage repair protein that mediates antioxidant gene production important for GSH synthesis [36]. The decreased level of GSH can further exacerbate the positive thromboinflammitary feedback loop in immunothrobosis by potentiating platelet aggregation and activation, and elevating levels of D-dimer [36-38]. Just as low levels of GSH are associated with pro-inflammatory states, increasing levels of GSH has been shown to decrease inflammatory states by mechanisms like reducing IL-6 and TGF-*β* in HIV patients [12,39,40]. GSH can also assist in the prevention of thrombi formation by assisting macrophages in removing fibrin [41]. Therefore, GSH may serve as an adjunctive therapy for COVID-19 [11].

## 5. COVID-19 Immunothrombosis Induction

Viruses have been shown to induce oxidative stress by depleting GSH [42]. A major characteristic of SARS-CoV-2 infection is oxidative stress, which leads to inflammation and vascular dysfunction. SARS-CoV-2 potentiates all three components of Virchow’s triad—hypercoagulability, blood flow stasis, and endothelial cell damage—to increase risk of thrombosis [43]. Wichmann *et al.* [44] found high incidence of thromboembolic events in complete autopsies from COVID-19 patients at an academic medical center in Germany. Of note, venous thromboembolism, arterial thrombosis, and microvascular thrombosis have all been described as complications of COVID-19 [45]. In addition, D-dimer levels were significantly elevated with increasing severity of COVID-19 [38].

**Fig. 2.**
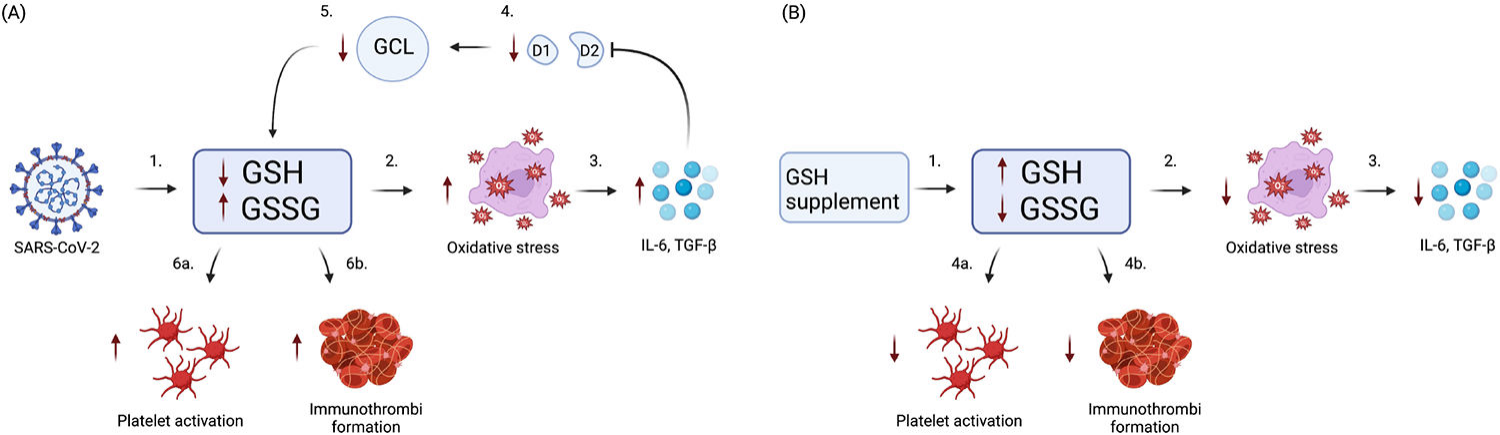
Role of GSH in COVID-19 Immunothrombosis. (A) SARS-CoV-2 infection causes GSH deficiency, eventually leading to platelet activation and immunothrombi formation. (1) Initially, SARS-CoV-2 reduces GSH and increases GSSG. (2) reduction in GSH and increase in GSSG causes an increase in oxidative stress. (3) In turn, this elicits the release of pro-inflammatory cytokines IL-6 and TGF-*β*. (4) These cytokines suppress the enzymes iodothyronine deiodinases type I (D1) and type II (D2). (5) Reduced D1 and D2 impair glutamate cysteine ligase (GCL), the rate-limiting enzyme in the GSH synthesis pathway. (6) This results in a repeating cycle of depleted GSH and increased GSSG, causing (6a) increased platelet activation, and (6b) increased immunothrombi formation. (B) Supplementation with GSH increases GSH, resulting in decreased platelet activation and immunothrombi formation. (1) GSH supplement increases GSH and decreases GSSG. (2) The increase in GSH and decrease in GSSG reduce oxidative stress. (3) Reduced oxidative stress results in decreased IL-6 and TGF-*β*. (4) The increase in GSH and decrease in GSSG ultimately (4a) reduce platelet activation and (4b) immunothrombi formation.

Research has shown that GSH, the most abundant antioxidant, is deficient in COVID-19 [8,37,38,46]. Oxidative stress leads to activation of pro-inflammatory cytokines [47]. Increased amounts of cytokines like IL-6 and TGF-*β* block enzymes that are contributory to the synthesis of GSH, exacerbating the GSH depletion [36]. This increases platelet activation, platelet aggregation, and risk of thrombosis in oxidative stress-related diseases, as seen in Fig. 2 [33,36]. The depletion of GSH is a major factor in the pathology of COVID-19, particularly in association with more severe manifestations [48]. A low baseline GSH level was the most common factor among patients with COVID-19 and risk factors identified to be associated with a high mortality rate including age, hypertension, ischemic heart disease, diabetes, and chronic respiratory disease [49]. Low GSH levels may serve as a biomarker for the risk of developing severe COVID-19, severe lung damage, and disease course of COVID-19 [46].

The main structural components of SARS-CoV-2 are the S protein, membrane, envelope, and nucleocapsid proteins. The S protein is responsible for viral entry into host cells via the ACE-2 receptor [50]. The S1 spike protein of SARS-CoV-2 binds to ACE-2 receptors, allowing the S2 spike protein to facilitate fusion of the viral membrane with the host cell membrane [51]. Higher rates of viral fusion into the host cells of those with comorbidities leads to significantly higher risk of morbidity and mortality [52]. Zhang *et al.* [53] found that SARS-CoV-2 induced platelet activation, aggregation, and dense granule release via the binding of the S protein to the ACE2 receptor. Ryu *et al.* [54] found that the addition of purified, recombinant SARS-CoV-2 S1 protein to a coagulation-competent normal plasma is sufficient to induce formation of anomalous clots that are resistant to fibrinolysis. These clots are identical to clots that have been found in Long COVID-19 patients and have been postulated to block capillaries, causing symptoms such as breathlessness, coagulopathies, and inflammation [6].

The other structural components of SARS-CoV-2, particularly the nucleocapsid protein, may play a role in inflammation. The nucleocapsid proteins of SARS-CoV-2 stimulate the release of IL-6 in a dose-dependent manner [55,56]. As mentioned before, this cytokine can decrease GSH and result in a cycle of GSH depletion and pro-inflammatory cytokine production [36]. IL-6 is associated with the cytokine storm that occurs in severe infections [11].

Lage *et al.* [8] found that markers associated with inflammatory responses persisted in COVID-19 patients after recovery, which was depicted as 52 days after infection onset. Particularly, the intracellular GSH levels were still found to be reduced compared to healthy control individuals. This suggests that decreased GSH not only contributes to acute COVID-19 but may also play a role in Long COVID.

There are varying results on effective therapies for COVID-19-induced thrombosis. In a few studies, early anticoagulation therapy in acute COVID-19 has been shown to improve patient outcomes, when compared to patients without any anticoagulation therapy [57-61]. Between patients who have received usual-care pharmacologic thromboprophylaxis and those who received therapeutic dose prophylactic anticoagulation, the INSPIRE, ACTION, and REMAP-CAP trials showed no difference in clinical outcomes [62]. Major bleeding occurred in a higher percentage of patients assigned to therapeutic-dose anticoagulation compared to those assigned to usual-care pharmacologic thromboprophylaxis [62].

A study of COVID-19 hospitalized patients showed that compared with patients who did not receive antiplatelet therapy, patients receiving acetylsalicylic acid had a significantly lower cumulative incidence of in-hospital death [45]. Another study found that antiplatelet therapy was associated with lower mortality rates, when compared to patients with no antiplatelet therapy and no anticoagulation therapy [63]. However, the RECOVERY trial found that patients treated with acetylsalicylic acid were not associated with reductions in mortality but had a slightly shorter duration of hospitalization and a higher proportion of these patients were discharged from the hospital alive within 28 days. Furthermore, the allocation to acetylsalicylic acid was associated with an increased risk of major bleeding and a decreased risk of thromboembolic complications [64]. In all, it appears that an ideal treatment would target thrombosis or platelet aggregation, without increasing the risk for bleeding. Findings suggest an oxidative stress pathway as a potential target for host-directed therapy to mitigate COVID-19 hyperinflammation and associated sequelae. Horowitz *et al.* [65] describes the use of GSH therapy in relieving dyspnea associated with COVID-19 pneumonia in two patients. It is likely that GSH supplementation, via a macrophage-induced pathway, plays a critical role in the removal of fibrin clots, which will be discussed later.

## 6. Role of IL-6 in Immunothrombosis

Interleukins are cytokines released by leukocytes and other types of cells in the body in response to biological threats [66]. Interleukins function as modulators for growth, differentiation and activation of inflammatory and immune responses due to their anti- and pro-inflammatory inherent capabilities [67]. While many interleukins exist, we will focus on IL-6, a proinflammatory cytokine which induces oxidative stress and systemic inflammation [68], and its effects on GSH. IL-6 expression is often strictly controlled at transcriptional and post-transcriptional levels, yet the dysregulated continual synthesis of IL-6 can contribute to pathological effects of tissue injury and hyperinflammatory states [69]. As previously discussed, the formation of thrombosis in hyperinflammatory state is influenced by the level of GSH, and recent studies have shown that GSH level is negatively affected by increased levels of IL-6 and TGF-*β* [12,39,40]. Valdivia *et al.* [12] demonstrated that in HIV-positive patients, the level of IL-6 significantly increased while the levels of free radicals increased due to significant reduction of GSH levels, and the introduction of liposomal GSH (L-GSH) was able to significantly increase the levels of GSH in the patients CD4+ T cells. The connection between lower level of GSH and the dysregulation of leukocyte-associated cytokines in HIV-positive plasma samples was also established, suggesting a correlation between inflammatory state and GSH level, and subsequent decrease in the levels of free radicals and immunosuppressive cytokines followed supplementing subjects with L-GSH [39]. However, the studies mentioned have not indicated the correlation between IL-6 and GSH levels in COVID-19 specifically.

Recently, the SARS-CoV-2 viral N protein, a nucleocapsid protein [70] was shown to upregulate the production of IL-6 via increased activation of its promoter in A549 human lung cells in a dose-dependent manner [36,55,56]. Other studies emphasize this connection by showing positive correlation between elevated IL-6 level and increased severity of COVID-19, such as exacerbated respiratory failure, hypercytokinemia and rapid progression to acute respiratory distress syndrome (ARDS) [11,71]. The increase in proinflammatory cytokines is hypothesized to deplete GSH to facilitate replication of SARS-CoV-2 and other viruses, leading to exacerbated symptoms [36]. The current proposed mechanism through which viruses such as SARS-CoV-2 accelerate their own replication is via the reduction of GSH level and increasing the production of reactive oxygen species [36]. Evidence supports that IL-6 has an ability to suppress the enzymes iodothyronine deiodinases type I (D1) and II (D2), reducing the conversion of prohormone thyroxine T4 to its active form T3, which ensures the deregulation of glutamate cysteine ligase (GCL) and the successive reduction of GSH synthesis [72,73]. The focal point drawn from all these studies combined suggests the formation of a repeating cycle of GSH depletion, leading to increased levels of IL-6 and TGF-*β* in the state of inflammation seen in COVID-19 and HIV-positive patients.

The relationship between IL-6 and formation of thrombosis was assessed in a study measuring levels of IL-6 in patients with deep vein thrombosis (DVT). Zhang *et al.* [74] found that IL-6 expression was increased while miR-338-5p, a small segment of non-coding RNA, was decreased in patients with DVT, suggesting a negative correlation. They were able to replicate the negative correlation in *murine* models where miR-338-5p knockdown increased IL-6 expression [74]. Although the exact mechanism is unclear, this finding is consistent with the suggestion that thrombosis is also inflammatory-mediated. Senchenkova *et al.* [75] also shows the relationship between IL-6 and abnormalities in platelet production, where thrombocytosis response, platelet hyperreactivity, and accelerated thrombus development were absent in IL-6-deficient mice.

## 7. Role of TGF-*β* in Immunthrombosis

TGF-*β* is a cytokine that has evolved to perform a regulatory role in expanding the system of epithelial and neural tissues along with activating the immune system and tissue repair [76]. TGF-*β* has potent regulatory and antiinflammatory activity, specifically serving as a critical regulator of thymic T-cell development and a crucial player in peripheral T-cell homeostasis and differentiation during immune response [77,78]. Like IL-6, TGF-*β* has been shown to be elevated in HIV-positive patients, representing a correlation between TGF-*β* elevation and the decrease of GSH levels, putting patients at similarly at risk for potent progression of SARS-CoV-2 virus and the formation of immunothrombosis [12,36,39]. Liu *et al.* [79] showed that TGF-*β* functions as a down-regulator of the expression of the enzyme GCL, the rate-limiting step enzyme involved in the synthesis of GSH as mentioned earlier, which negatively effects the production of GSH. Another study by Liu *et al.* [80] suggests a potential relationship between increased expression of TGF-*β* and decreased level of GSH in TGF-*β*-mediated fibrogenesis, thus leading to increasing level of ROS. Similarly, as seen with IL-6, TGF-*β* is a potent inhibitor of GSH synthesis in the lung epithelial cell line A549, with Arsalane *et al.* [81] demonstrating complete depletion of GSH 72 hours after exposure to exogenous TGF-*β*1. The author of this study hypothesized that the TGF-*β*1 mediated reduction in GSH synthesis is associated with the decrease in both *γ*-GCS protein and the levels of mRNA expression that codes for *γ*-GCShs, both of which are essential for maintaining the adequate level of endogenous GSH [81].

## 8. Role of D-Dimer in Immunothrombosis

D-dimer is a protein fragment found in the plasma when a blood clot undergoes degradation by fibrinolysis. Although D-dimer can exist at low levels in the plasma of healthy individuals from the physiologic breakdown of fibrin, elevated levels develop in a number of pathologic conditions [82]. Levels >500 ng/mL are frequently used to denote a “positive” test result [83,84]. Venous thromboembolism (VTE), cancer, and pneumonia were frequently present when ultra-high plasma D-dimer levels were encountered, and mortality was high when the levels were >15,000 ng/mL [85].

A state of oxidative stress is associated with diabetes, aging, cancer, and COVID-19, among others, and result in GSH depletion. The increased ROS level impacts the integrity of the RBC membrane, which impacts the red blood cell (RBC) function, leading to impaired hemostasis and thrombosis. The RBC aggregation that results leads to a hypercoagulable state [86]. The endothelial cell lining also becomes dysfunctional in a state of increased ROS, triggering platelet adhesion and activation. This phenomenon can be seen in aging, which is characterized by an overproduction of ROS [86]. It also presents with a higher incidence of thromboembolism and venous thrombosis [87]. Erythrocyte oxidation stress leads to thrombotic events in these conditions, marked by an elevated D-dimer. This suggests an important relationship between GSH and D-dimer levels.

Nwose *et al.* [88] found a statistically significant lower level of GSH and statistically significant higher D-dimer in diabetic and pre-diabetic patients compared to controls. There was also a significantly negative correlation between GSH and D-dimer levels in diabetic and pre-diabetic patients [88]. This finding can be attributed to erythrocyte oxidative stress induced by hyperglycemia which depletes GSH.

Elevated D-dimer is a well-studied biomarker for disease severity and mortality in COVID-19 [89]. Yao *et al.* [38] found that D-dimer elevation was present in 74.6% of patients in Remnan Hospital of Wuhan University, Wuhan, China, and was the only variable associated with increased mortality odds. Shah *et al.* [89] also found an association between elevated D-dimer levels and COVID-19 severity, with 15% of recovered COVID-19 patients retaining persistently elevated D-dimer level after a median of 3 months following infection. Kryukov *et al.* [46] found a negative association between D-dimer levels and GSH levels, and identified an association between low total GSH and risk of severe COVID-19. This observation is secondary to the elevated incidence of thromboembolic complications in COVID-19 patients. There currently exists a lack of diagnostic method to accurately determine the presence of microemboli. As such, testing for D-dimer may serve as a method of guiding clinical suspicion and monitoring response to therapy.

## 9. Role of Macrophages in Immunothrombosis

Macrophages have a wide variety of functions, both in physiological processes and in disease pathogenesis. Macrophages have immunological function in bacterial, viral and parasitic infections, and they also function in inflammatory and hemostatic processes [90,91]. The role of macrophages in immunothrombosis is thought to be related to their role in fibrinolysis. Degradation of fibrin clots is mostly associated with the conversion of plasminogen by tissue-plasminogen activator to a serine protease plasmin. The degradation of fibrin by plasmin and the deposition of fibrin products is a process that has been well described, however, there remains a less studied pathway in which extravascular fibrin deposits are ultimately removed and degraded [92]. The clearance of fibrin from the blood has been viewed to involve the phagocytosis of microclots, presumably by macrophage [93]. The clearance mechanism of which macrophage remove fibrin has been closely tied to the activity of plasminogen and plasmin. It has been shown that once activated, plasmin activates matrix metalloproteinases that allows for macrophage movement through the extracellular matrix [94]. In several instances, the deficiency of plasminogen has shown to cause a decrease in the recruitment of macrophages [94,95]. Beyond macrophage migration, it has also been shown the lack of plasmin activity leads to the inability of macrophage to clear fibrin deposits [95]. This process has further been demonstrated by a study from Motley *et al.* [92] that describes a novel pathway of macrophage endocytosis of fibrin. They found that there is a specific macrophage population, CCR2 positive, that is responsible for endocytosis of fibrin and elimination of these cells results in decreased cellular fibrin uptake. Furthermore, these macrophages are morphologically distinct from collagen degrading macrophages. They also confirmed the findings of previous studies in implicating plasmin/plasminogen in the function of macrophagic endocytosis of fibrin, noting that plasminogen is needed for the fragmentation of fibrin to expose cellular binding sites for endocytic uptake but also that plasmin directly stimulates macrophage phagocytosis. In a model of plasminogen-deficient mice, they showed that there was a reduction in endocytosis of fibrin, suggesting that leukocytic fibrinolytic pathways are largely, although not entirely, dependent on plasminogen [92]. Similarly to the loss of plasminogen, the loss of enzymatic activity of plasmin had similar findings of reduced fibrin endocytosis by leukocytes, further demonstrating the need for plasmin cleavage of fibrin prior to cellular uptake. Following endocytosis, degradation of fibrin occurs within lysosomes of macrophage. This process involves the binding of the amino-terminus alpha chain of fibrin to cell surface receptors on macrophage [90]. Beyond the direct role of macrophage in fibrin clot removal, macrophage and other phagocytes have also been shown to modulate thrombosis through the removal of active coagulation factors and activated platelets thus decreasing the ability for thrombus formation [96].

The interaction between macrophages and GSH relates both to the function of GSH as the main antioxidant of ROS and its role of signaling within the innate immune system [36]. Failure of detoxification will result in ROS reacting with cellular components and ultimately leading to impaired cellular function [11]. Control of infections such as *Mycobacterium tuberculosis* (*M. tb*) are dependent upon macrophages and their ability to inhibit pathogen growth with ROS [11]. Individuals with HIV and type 2 diabetes mellitus (T2DM) have shown to have decreased levels of GSH due to the increased production of free radicals depleting GSH [97,98]. This subsequently leads to impaired macrophage productivity and decreased control of *M. tb* infection in these individuals [12,99]. Increased *M. tb* survival in these patients can also be related to the role of GSH in the signaling of the innate immune system. IFN-gamma is responsible for the activation of macrophages, enhanced antigen presentation, and the induction of nitric oxidated mediated killing mechanisms [99]. Individuals with T2DM or HIV have been shown to have decreased levels of IFN-gamma. Supplementation of these individuals with L-GSH has shown to significantly increase production of IFN-gamma and decrease levels of IL-10. The combined efforts of increased macrophage activation from IFN-gamma and reduced inhibition from IL-10 leads to increased control of *M. tb* infection in these patients [12,99].

Therefore the role of macrophages in immunothrombosis appears to be both in disease control as well as resolution of immunothrombosis through fibrin clot degradation. The function of macrophages within these processes is tightly connected to the levels of GSH. It would be expected then that supplementation of GSH in patients would enhance the productivity of macrophage both in disease control to prevent immunothrombosis and in increased clot dissolution in immunothrombosis.

## 10. Proposed Mechanism on the Role of GSH in COVID-19 Immunothrombosis

There is sufficient evidence that SARS-CoV-2 infection decreases GSH, but the exact mechanisms by which this occurs remains unclear. Bartolini *et al.* [100] demonstrated that SARS-CoV-2 lowered uptake of the GSH precursor Cys and increased efflux of thiols, effectively lowering GSH by impairing metabolism of cellular GSH. In addition, they found that blocking viral replication successfully prevented GSH depletion [100]. SARS-CoV-2 also inhibits nuclear factor-erythyroid 2 p45-related factor 2 (Nrf2), a primary transcription factor critical in increasing expression of glutamate cysteine ligase (GCL) [101-103]. As mentioned previously, COVID-19 also depletes GSH through intracellular radical generation and inhibition of BRCA1. Impaired metabolism coupled with increased consumption of GSH results in decreased levels of GSH and increased levels of GSSG. GSH may also be reduced prior to SARS-COV-2 infection as reduced levels are seen in old age, other inflammatory conditions, and vitamin D deficiency, representing significant risk factors for severe SARS-CoV-2 infection [101].

**Fig. 3.**
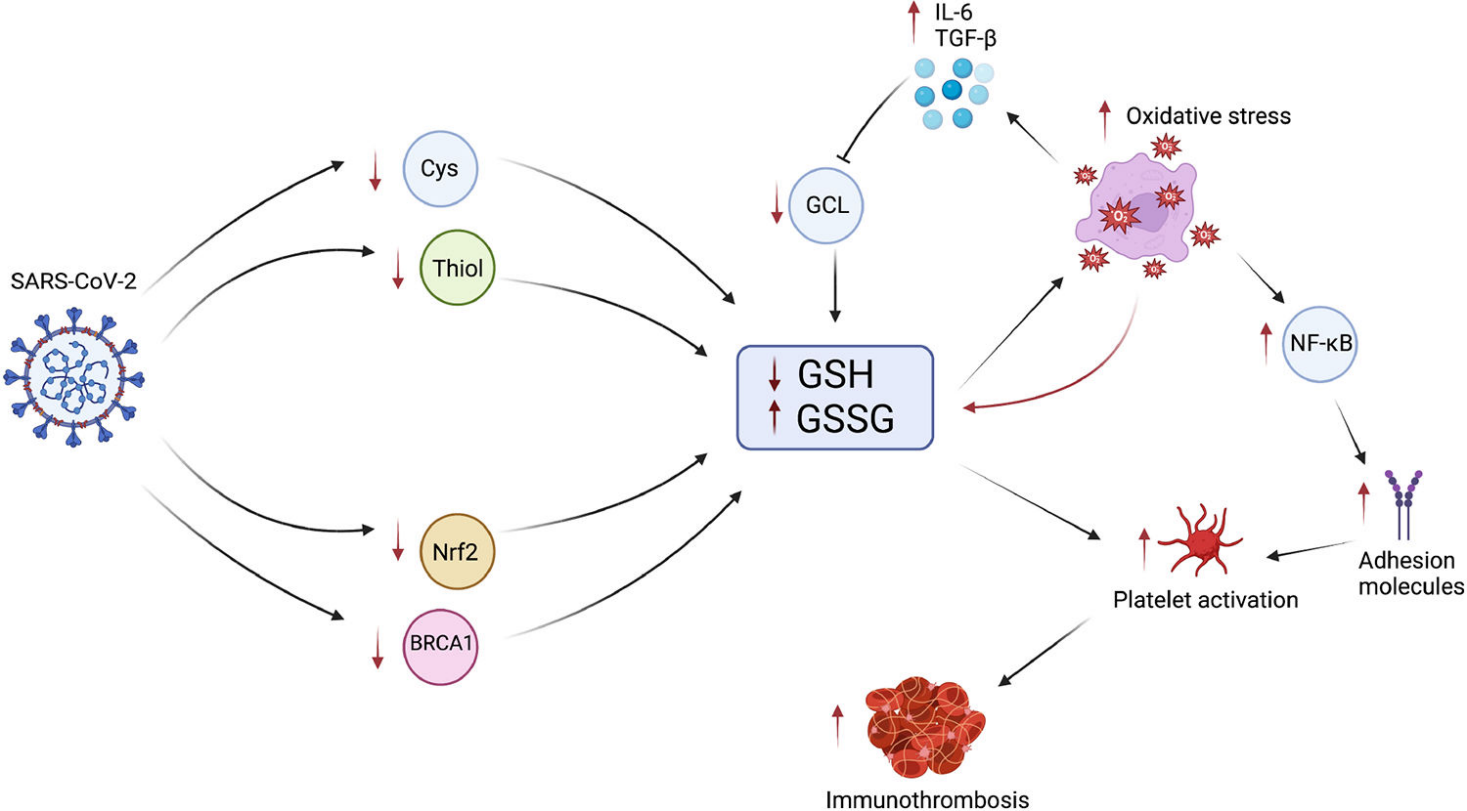
The Role of GSH in COVID-19 Immunothrombosis. Infection with SARS-CoV-2 results in decreased intracellular Cys and thiol due to decreased Cys uptake and increased thiol efflux, reducing GSH synthesis. Infection also inhibits Nrf2 and BRCA1, further reducing GSH synthesis. The reduction in GSH and increase in GSSG results in impaired ability to control oxidative stress. Increased oxidative stress further propagates the biochemical cascade by consuming additional GSH, increasing the production of cytokines IL-6 and TGF-*β*, as well as activating NF-*κ*B. Increasing levels of IL-6 and TGF-*β* impair GCL, further reducing GSH synthesis. NF-*κ*B increases the production of prothrombotic adhesion molecules which increase platelet activation. Increased GSSG and decreased GSH also contribute to further platelet activation. Together, these events lead to the increased production of immunothrombosis in COVID-19.

Decreased GSH potentiates increased oxidative stress and increased release of cytokines IL-6 and TGF-*β*. Increased IL-6 suppresses D1 and D2, downregulating GCL. Increased levels of TGF-*β* have also been shown to reduce GCL and GSH synthesis. This feedback loop creates a cascade further depleting GSH levels and increasing GSSG. The decreased levels of GSH and increased oxidative stress trigger endothelial damage, decreasing endothelial NO, which activates NF-*κ*B, ultimately increasing production of prothrombotic adhesion molecules. Decreased GSH and increased GSSG trigger platelet activation, aggregation, and immunothrombosis formation, as seen in Fig. 3. Activated platelets release plateled-derived ROS, which further contribute to the cycle of platelet activation, adhesion, and recruitment. Conversely, supplementation with GSH has been shown to reverse this biochemical cascade by reducing IL-6 and TGF-*β*, inhibiting platelet activation and aggregation, and reducing thrombus formation. In addition to its biochemical protective role, more research is arising demonstrating GSH is capable of suppressing SARS-CoV-2 spike-mediated cell-cell fusion and syncytium formation, potentially serving to terminate the cascade before it begins [104].

## 11. Conclusions

Immunothrombosis is an important pathological feature of inflammatory conditions and infections like COVID-19. While immunothrombosis can serve as a natural defense mechanism by immune cells like neutrophils and monocytes, dysregulated immune responses can create a thrombotic cascade. Excessive cytokine release seen in COVID-19 can cause inflammation of endothelial cells, resulting in damage and release of TF in pulmonary microvessels, yielding microclots. These microclots can block capillaries, creating a hypoxic environment which furthers the coagulation cascade, or they can remain as emboli and circulate in the blood. Patients who experience severe acute- or long-COVID-19 infection are at an increased risk of adverse thrombotic events due to the hypercoagulable state. Diagnosing the presence of microclot formation from immunthrombosis in late-COVID-19 proves challenging, as current diagnostic modalities for evaluating the presence of a thrombus are not sufficient for identifying the circulating microclots and those embedded in capillary beds. The presence of D-dimers in blood may serve as an important biomarker in both monitoring infection course and estimating long-term adverse thrombosis risk.

Research has previously evaluated the role of GSH in the maintenance of ROS and its association with cytokine levels in states such as HIV and diabetes, but had not yet evaluated GSH depletion as a dominant cause of immunothrombosis in COVID-19. SARS-CoV-2 has been shown to decrease GSH by reducing Cys uptake, increasing thiol efflux, inhibiting Nrf2, and inhibiting BRCA1. There is sufficient evidence that there is a negative correlation between the cytokines IL-6 and TGF-*β* and GSH. There is also sufficient evidence that COVID-19 infection induces proliferation of IL-6 and TGF-*β*, as well as evidence of low GSH in patients with COVID-19 infection. Likewise, there is evidence that increasing levels of GSH through supplementation, like L-GSH, is able to reduce levels of IL-6 and TGF-*β*. In addition to the role of cytokines in immunothrombosis, macrophages have proven an important role in fibrin degradation. There is sufficient evidence that in inflammatory states like HIV and diabetes, excessive ROS can deplete GSH and reduce macrophage productivity. There is also a correlation between low levels of IFN-gamma, a cytokine responsible for macrophage activation and enhancement, and these states. Evidence shows that supplementation with GSH can increase IFN-gamma and decrease IL-10 to improve macrophage response and subsequently, fibrin removal. There is sufficient evidence to show that depletion of GSH in platelets results in increased platelet adhesion and aggregation, and that exogenous GSH supplementation may inhibit platelet activation. There is also evidence that enzymes regulating GSH, GTX, serve protective roles against platelet hyperactivity and thrombosis. In addition to platelet modulation, decreased levels of GSH increases local ROS in endothelial tissue contributing to a decrease in NO, an upregulation of NF-*κ*B, and subsequent increase in cytokine production and prothrombotic adhesion molecules. With enough evidence demonstrating the occurrence of immunothrombosis in COVID-19 and other inflammatory conditions, as well as a clear association between deficient GSH and mediators of thrombosis and immunothrobosis, GSH may reside at the top of the cascade of biochemical events to reduce immunothrombosis. Given these associations, GSH warrants further research as a novel therapeutic and risk-assessment biomarker.
